# Molecular Characterization of Severin from *Clonorchis sinensis* Excretory/Secretory Products and Its Potential Anti-apoptotic Role in Hepatocarcinoma PLC Cells

**DOI:** 10.1371/journal.pntd.0002606

**Published:** 2013-12-19

**Authors:** Xueqing Chen, Shan Li, Lei He, Xiaoyun Wang, Pei Liang, Wenjun Chen, Meng Bian, Mengyu Ren, Jinsi Lin, Chi Liang, Jin Xu, Zhongdao Wu, Xuerong Li, Yan Huang, Xinbing Yu

**Affiliations:** 1 Department of Parasitology, Zhongshan School of Medicine, Sun Yat-sen University, Guangzhou, People′s Republic of China; 2 Key Laboratory of Tropical Diseases Control at Sun Yat-sen University, Ministry of Education, Guangzhou, People′s Republic of China; McGill University, Canada

## Abstract

**Background:**

Clonorchiasis, caused by the infection of *Clonorchis sinensis* (*C. sinensis*), is a kind of neglected tropical disease, but it is highly related to cholangiocarcinoma and hepatocellular carcinoma (HCC). It has been well known that the excretory/secretory products of *C. sinensis* (*Cs*ESPs) play key roles in clonorchiasis associated carcinoma. From genome and transcriptome of *C. sinensis*, we identified one component of *Cs*ESPs, severin (*Cs*severin), which had three putative gelsolin domains. Its homologues are supposed to play a vital role in apoptosis resistance of tumour cell.

**Methodology/Principal Findings:**

There was significant similarity in tertiary structures between human gelsolin and *Cs*severin by bioinformatics analysis. We identified that *Cs*severin expressed at life stage of adult worm, metacercaria and egg by the method of quantitative real-time PCR and western blotting. *Cs*severin distributed in vitellarium and intrauterine eggs of adult worm and tegument of metacercaria by immunofluorence assay. We obtained recombinant *Cs*severin (r*Cs*severin) and confirmed that r*Cs*severin could bind with calciumion in circular dichroism spectrum analysis. It was demonstrated that r*Cs*severin was of the capability of actin binding by gel overlay assay and immunocytochemistry. Both Annexin V/PI assay and mitochondrial membrane potential assay of human hepatocarcinoma cell line PLC showed apoptosis resistance after incubation with different concentrations of r*Cs*severin. Morphological analysis, apoptosis-associated changes of mitochondrial membrane potential and Annexin V/PI apoptosis assay showed that co-incubation of PLC cells with r*Cs*severin *in vitro* led to an inhibition of apoptosis induced by serum-starved for 24 h.

**Conclusions/Significance:**

Collectively, the molecular properties of *Cs*severin, a molecule of *Cs*ESPs, were characterized in our study. r*Cs*severin could cause obvious apoptotic inhibition in human HCC cell line. *Cs*severin might exacerbate the process of HCC patients combined with *C. sinensis* infection.

## Author Summary

*Clonorchis sinensis* (*C. sinensis*) has afflicted more than 35 million people in world and approximately 15 million in China, creating a socio-economic burden in epidemic regions. The infection of *C. sinensis* is highly related to cholangiocarcinoma and hepatocellular carcinoma (HCC). It has been documented that excretory/secretory products of *C. sinensis* (*Cs*ESPs) involved in the pathogenesis of HCC. *Cs*severin, expressed at life stage of egg, metacercaria and adult worm, was a component of *Cs*ESPs. In the current study, we characterized the properties of *Cs*severin such as sequence signature, actin and calciumion binding activity. In addition, we demonstrated that *Cs*severin could cause apoptotic inhibition in spontaneously apoptotic human HCC cell line PLC cells by using morphological analysis, detection of the apoptosis-associated change of mitochondrial membrane potential (MMP) as well as Annexin V/PI apoptosis assay. Our study provided an exploratory sight view of mechanism involved in progress of carcinoma associated with the infection of *C. sinensis* and *Cs*severin might exacerbate the process of *C. sinensis* infected HCC patients.

## Introduction

*Clonorchis sinensis* (*C. sinensis*) has been proven to be the causative agent of clonorchiasis, which is endemic in China, Korea and Vietnam [1], [2], [3]. As an important food-borne parasite, *C. sinensis* has afflicted more than 35 million people in world and approximately 15 million in China, creating a socio-economic burden in epidemic regions [4]. Most clonorchiasis cases are due to the consumption of raw freshwater fish containing infective *C. sinensis* metacercariae, which excyst in the duodenum until they grow into juvenile *C. sinensis* and then migrate into the bile ducts of their host [5], [6]. Both experimental and epidemiological evidence have implied that long-term infections with liver flukes lead to chronic pathological changes, including hepatomegaly, hepatic fibrosis, cholangitis, cholecystitis, adenomatous hyperplasia, and cholangiocarcinoma (CCA) [7], [8], [9]. Furthermore, *C. sinensis* was recently classified along as a Group I biological carcinogen by the World Health Organization [10], [11]. In endemic area of China, 16.44% of HCC patients were infected with *C. sinensis*, while 2.40% were infected in non-tumor patients. The OR value and 95% CI in HCC group were 8.00 and 4.34–14.92 [12], [13], [14], so that we should pay high attention to the relationship between primary hepatocellular carcinoma and the infection of *C. sinensis*. It has been well known that the excretory/secretory products of *C. sinensis* (*Cs*ESPs) can cause histopathological changes such as bile duct dilatation, inflammation and fibrosis, and adenomatous proliferation of the biliary epithelium [15]. In the present studies, from the published genome [16] and transcriptome [17], [18] of *C. sinensis*, we identified one component of *Cs*ESPs, *Cs*severin, which has three putative gelsolin domains.

The gelsolin superfamily is conserved in mammalian as well as in non-mammalian organisms and takes the leading role in controlling actin organization or actin filament remodeling. The family has some specific and apparently non-overlapping particular roles in several cellular processes, including cell motility, control of apoptosis and regulation of phagocytosis [19]. Initial evidence of anti-apoptotic effect of gelsolin was provided by the observation that a point mutation in mouse gelsolin confers on this protein tumor-suppressor activity against H-ras oncogene transformed NIH-3t3 cells [20], [21]. Direct evidence of the inhibitory role of gelsolin was provided by Ohtsu et al., who generated Jurkat transfectants expressing up to threefold gelsolin than wild-type cells. These transfectants exhibited a phenotype more resistant to apoptosis induced by several stimuli [22]. Moreover, it has been reported that human cytoplasmic gelsolin can prevent apoptotic mitochondrial changes such as mitochondrial membrane potential loss by binding to mitochondrial voltage-dependent anion channel (VDAC) [23].

Large-scale gene sequencing efforts have revealed gelsolin homologues in the majority of parasitic phyla [24], [25], [26], [27], [28]. In the current study, we presented for the first time the molecular characteristics of *Cs*severin. We described the detection of recombinant *Cs*severin (r*Cs*severin) binding to cytoskeletal actin filaments of human hepatocarcinoma PLC cells and investigated its potential anti-apoptotic role on PLC cells as an ingredient of *Cs*ESPs *in vitro*. The present study is a cornerstone for researches on biological characterization of *Cs*severin in the future. In addition, our work will provide an exploratory sight view of mechanism involved in progress of carcinoma associated with the infection of *C. sinensis*.

## Materials and Methods

### Ethics statement

*C. sinensis* flukes were isolated from naturally infected cats (Guangdong Province, China) for sample preparation. Animals in experiments were all purchased from animal center of Sun Yat-sen University and raised carefully in accordance with National Institutes of Health on animal care and the ethical guidelines. All experimental procedures were approved by the animal care and use committee of Sun Yat-sen University (Permit Numbers: SCXK(Guangdong) 2009-0011).

### Cell culture

PLC and human normal hepatocyte L-02 cells were a gift from Dr. Wang Shutong and Dr. Xie wenxuan (the first affiliated hospital of Sun Yat-Sen University) and routinely cultured in high glucose DMEM medium (Gibco, USA) supplemented with 10% fetal bovine serum (Gibco, USA) and penicillin–streptomycin (100 units/ml) in 5% CO_2_ at 37°C. Serum-starved PLC were prepared by incubating the cells in high glucose DMEM medium at 37°C and 5% CO^2^ with fetal bovine serum deprivation for at least 24 h.

### Sequence analysis of *Cs*severin

The gene (GenBank accession No. GAA30384.2) predicted encoding homologue of severin was screened from *C. sinensis* genome by blastx and Open Reading Frame (ORF) Finder program at NCBI (http://www.ncbi.nlm.nih.gov). The alignment of its deduced amino acid sequences with homologues from other species were analyzed and shown with Vector NTI. Proteomics bioinformatics tools such as Motif-Scan, InterPro-Scan and Swiss-Model were used to analyze the protein characteristics including physicochemical parameters, conserved domains and spatial structure. The phylogenetic tree was constructed online (http://www.ebi.ac.uk/Tools/clustalw/index.html).

### Preparation of anti-*Cs*severin IgG

The ORF of severin was amplified using the following primers: sense: 5′- ATAGGATCCATGCCGGAGTACT -3′(underlined, *BamH*I) and antisense: 5′- CGCAAGCTTTCATTCGAGAACC-3′ (underlined, *Hind* III). The PCR was carried out for 32 cycles at 94°C for 45 s, 51°C for 45 s, and 72°C for 45 s, and extension for 10 min at 72°C after the last cycle in a DNA-Thermal Cycler (Biometra, Germany). PCR products were purified and digested with *BamH*I and *Hind* III, and then subcloned into prokaryotic expression vector 6×His tag pET28a(+) (Novagen, Germany). After digestion with *BamH*I and *Hind* III, the recombinant plasmid was confirmed by DNA sequencing and then transformed into *E. coli*, BL21 (Promega, USA). The expression of r*Cs*severin was induced by 1 mM isopropyl-β-D-thiogalactopyranoside (IPTG) for 5 h at 37°C. After induction, the bacteria were harvested by centrifuging at 4°C for 15 min at 8,000×g and suspended in lysis buffer (0.5 M NaCl, 20 mM Tris–HCl, 5 mM imidazole, pH 8.0), sonicated on ice, and centrifuged at 10,000×g for 15 min at 4°C. The fusion protein was batch-purified using His Bind Purification kit (Novagen, USA) and the eluted fractions containing r*Cs*severin were pooled and dialyzed with phosphate-buffered saline (10 mM phosphate buffer, 27 mM KCl, 137 mM NaCl, pH 7.4). Protein samples were subjected to 12% sodium dodecyl sulfate polyacrylamide gel electrophoresis (SDS-PAGE) and visualized by Coomassie brilliant blue G-250, the concentration was measured by a Bicinchoninic acid assay (BCA, Novagen, USA) according to manufacturer's instructions. Then, 100/50 µg of r*Cs*severin were mixed with an equal volume of incomplete Freund's adjuvant and injected subcutaneously to six-week-old male Sprague-Dawley (SD) rats (purchased for experiments under the Guide for the Care and Use of Laboratory Animals). Boost injections were given at 2 and 5 weeks after first injection. Anti-serum was collected at 1 week after the second booster, then aliquoted and stored in −80°C. Sera from naïve rats were also collected for using as control.

### Western blotting

*Cs*ESPs and sera from *Cs*ESPs immunized rat were obtained by referring to previous study [29]. 10 µg of r*Cs*severin or *Cs*ESPs were subjected to 12% SDS-PAGE and transferred to polyvinylidene fluoride (PVDF) membranes. Successively, the membranes were blocked with 1% bovine serum albumin in phosphate-buffered saline (PBS) overnight at 4°C, washed five times with PBS-0.05% Tween 20 (PBS-T, pH 7.4), and incubated with His-tag monoclonal antibody, sera from naïve rats, r*Cs*severin immunized rats, *C. sinensis*-infected rats or *Cs*ESPs immunized rats (1∶100 dilutions) followed by HRP-conjugated goat anti-mouse/rat IgG (Proteintech; dilution of 1∶2,000) at 37°C for 2 h. After adequately washing with PBS-T, the membrane was incubated with horseradish peroxidase (HRP)-conjugated goat anti-rat IgG in 1∶2000 dilutions (Proteintech, USA) at 37°C for 1 h. Detection was then carried out by enhanced chemiluminescence (ECL) method.

### Expression level of *Cs*severin at life-stages of *C. sinensis*

Intact living adult worms were collected from biliary tracts of infected cats and washed extensively and gently in physiological saline to remove any contamination from hosts. Eggs and metacercariae were also collected as described previously [30], [31]. They were stored in sample protector (Takara) at −80°C for RNA/DNA extraction or 4% formaldehyde for immunofluorescence assay. Total RNA was extracted from each sample using TRIZOL reagent (Invitrogen, USA) according to manufacturer's instructions, and total RNA was treated with DNase (Promega, USA) to remove any contaminated DNA. Their total cDNA were obtained by the method of reverse transcription PCR by using Reverse Transcriptase XL (TaKaRa) and Oligo18 primer referred to the manuals. Severin RNA was detected with SYBR Premix Ex Taq Kit (TaKaRa, Japan) according to the manufacturer's protocol. Real-time PCR was conducted in the BIO-RADiQ5 instrument (BioRad, USA) using specific primers (sense: 5′-TACAGCACCGTGAAGTAGATGG-3′; antisense: 5′- CAGACCGTGACAGAGTAGCAGA-3′). β-actin from *C. sinensis* (GenBank accession No. EU109284) was used as an internal control [32], which was amplified with the primers (forward primer: 5′-ACCGTGAGAAGATGACGCAGA-3′, reverse primer: 5′-GCCAAGTCCAAACGAAGAATT-3′) designed by primer premier 5.0. The transcripts of *Cs*severin were detected using SYBR Premix Ex Taq Kit (TaKaRa, Japan) according to the manufacturer's protocol. PCR was carried out in a total volume of 20 µl, consisting of 2 µl cDNA, 10 µl SYBR Premix Ex Taq (2×), 0.4 µl Severin forward and reverse primer (10 µM), and 7.2 µl RNase-free distilled H_2_O. The real-time PCR program consisted of an initial denaturation step at 95°C for 30 s, 45 cycles of 95°C for 5 s, and 60°C for 20 s. The real-time PCR amplification was conducted in the BIO-RADiQ5 instrument (BioRad, USA). To complete the protocol, a melting curve was constructed using the following program: 95°C for 30 s, 65°C for 15 s, followed by increase to 95°C while continuously collecting fluorescence signal. Semiquantitative analysis as performed by the comparative 2^−ΔΔCt^ method [33].

The total proteins of adult worms, metacercariae, and eggs were respectively homogenized in RIPA lysis buffer (containing 1 mM proteinase inhibitor PMSF, Biotech, USA) followed by centrifugation at 10,000×g for 15 min. 20 µg of total proteins from each life cycle stage were separated on SDS-PAGE (12% gel) and electro-transferred onto PVDF membrane. The membrane was blocked with 1% bovine serum albumin in PBS overnight at 4°C, washed with PBS-T, and incubated with anti-*Cs*severin rat serum (1∶100 dilutions) or pre-immune rat serum (1∶100 dilutions) at 37°C for 2 h. After extensively washing with PBS-T, the membrane was incubated with HRP-conjugated goat anti-rat IgG in 1∶2000 dilutions (Proteintech, USA) at 37°C for 1 h. Detection was then carried out by ECL.

### Immunohistochemical localization of *Cs*severin in *C. sinensis* adults and metacercariae

Fresh adult worms and metacercariae of *C. sinensis* were fixed with 4% formaldehyde, embedded with paraffin wax, and sliced into 4-µm-thick sections. After dewaxing and dehydration, slides were blocked with goat serum overnight at 4°C, and incubated with anti-r*Cs*severin sera (1∶100 in 0.1% PBS-T) at room temperature for 2 h. Sera from naïve rats were used as a negative control. The slides were washed twice and incubated with goat anti-rat IgG labeled with red fluorescent Cyanine dye 3 (Cy3, Proteintech; 1∶400 in 0.1% PBS-T). Fluorescence microscopy was used in visualization of antibody staining.

### Circular Dichroism (CD) measurements

As the protein contains a potential Ca^2+^-binding domain, Ca^2+^-binding will change its conformation of secondary structure which can be detected by CD [34], [35], [36]. CD measurements were carried out on a J-810 Circular Dichroism Spectrometer (Jasco, Japan) with the Jasco Spectra Manager software at room temperature. Three samples were assayed: purified r*Cs*severin in PBS, purified r*Cs*severin in PBS containing 1 µM CaCl_2_, and purified r*Cs*severin in PBS containing 1 µM EDTA to remove combined Ca^2+^ during expression of r*Cs*severin in bacteria and purification in solutions. Secondary structure was analyzed using Jasco Spectra Manager Secondary Structure Analysis program. Far-UV CD spectrum was acquired using a 0.2 mm path length cell at 0.2 nm intervals over the wavelength range from 190 to 250 nm. Three scaning values were averaged for each sample and were corrected by subtracting buffer contribution from parallel spectra in the absence of *Cs*severin. The concentration of *Cs*severin was kept at 1 µM in 10 mM sodium phosphate buffer pH 7.4 and then the CD data were converted to molar units.

### Actin binding activity of r*Cs*severin

Gel overlay assay and immunocytochemistry were employed to investigate the actin binding activity of r*Cs*severin. F-actin (from rabbit muscle, 99% similar to human F-actin, Sigma-Aldrich) and its fragments digested with 0.25% trypsin (Sigma-Aldrich, USA) at 37°C for 1 h, were separated on 12% SDS-PAGE and electrophoretically transferred onto PVDF membranes. Membranes then were blocked with TBS-T (25 mM Tris-HCl, pH 7.2, 50 mM NaCl, 0.5% Tween-20) containing 5% BSA overnight at 4°C and washed (3 times, for 15 min each) in TBS-T. Then, membranes were incubated with 0.1 mg/ml r*Cs*severin in TBS-T for 1 h at room temperature. After washing extensively, membranes were incubated with anti-*Cs*severin rat serum (1∶100 dilutions) in TBS-T for 1 h at room temperature. The membranes were incubated with 1∶2000 HRP-conjugated secondary antibodies against rat IgG in TBS-T for 1 h at room temperature after washing. Following extensive washing in TBS-T, the membranes were at last incubated with diaminobenzidine substrate solution to develop color after washing again [37].

In immunocytochemistry assay, the PLC cells were seeded into sterile Petri dish (Nest, diameter of 15 mm) which is special for the detection of laser scan confocal microscopy, at a density of 2×10^4^ cells per well and then cultured for 24 h. The PLC cells were washed four times with PBS and then fixed with 2 ml of 4% paraformaldehyde solution in PBS at room temperature for 30 min, then treated with 50 mM NH_4_Cl for 10 min, to reduce aldehyde groups. The cells were permeabilized for 4 min at 4°C with 0.3% Triton X-100 in PBS. At the next step, cells were incubated in PBS buffer containing 3% of BSA for 1 h, followed by coated with r*Cs*severin overnight at 4°C. To visualize cytoskeleton, cells were incubated overnight at 4°C with mouse anti human F-Actin monoclonal antibody (AbD Serotec, UK) diluted 1∶1000, then subsequently incubated overnight at 4°C with rat anti-r*Cs*severin serum (1∶100) for 12 h at 4°C. The incubation with secondary antibodies was carried out at RT for 2 h, using fluorescein isothiocyanate (FITC)-conjugated goat anti-mouse IgG (Proteintech, USA) diluted 1∶200 and Cyanine dye 3 (Cy3)-conjugated goat anti-rat IgG (Proteintech, USA) diluted 1∶400 at the same time. All antibodies were diluted with 1% BSA in PBS buffer and all steps described above were preceded by intensive washes in PBS. After finally washing with water, cover dishes were mounted on slides with Hoechst 33258 (Sigma, USA). By contrast, to visualize whether r*Cs*severin could bind with cytoskeletal actin filaments *in vitro*, PLC cells were serum-starved overnight after incubating 24 h in standard conditions, and coated with r*Cs*severin in DMEM with 2% FBS for 48 h before fixed with 4% paraformaldehyde solution. The following steps were similar with that mentioned above previously. Images were finally obtained with the LSM 710 laser scanning confocal microscope (Zeiss).

### Apoptosis assays

After being induced spontaneous apoptosis by serum-starved for 24 h and treated with r*Cs*severin at different concentrations of 10, 20, 40, 80 µg/ml and PBS for 48 h, 1–5×10^5^ PLC cells were collected by centrifugation, and then incubated with Annexin V/propidium iodide (PI), provided by the Apoptosis Detection Kit (Lankebio, China). The cells were washed twice in PBS and resuspended in 500 µl of 1×Binding Buffer before being incubated with 5 µl of Annexin V and 10 µl of PI. The cells were then analyzed by using flow cytometry after incubation for 5–10 min in dark. Early apoptotic cells were stained with AnnexinV alone whereas necrotic and late apoptotic cells were stained with both Annexin V and PI.

PLC cells (5×10^4^ cells per well) were seeded into a 6-well culture plate and cultured as described above. After treatment with Apoptosis Inducers (Beyotime, Chain), the cells were washed twice with PBS, permeabilized with 0.3% Triton in PBS, and stained with Hoechst 33258 for 5 min in dark. Morphologic changes in apoptotic nuclei were observed and photographed under the inverted fluorescence microscope (Leica DMI4000B, Germany) with emission wavelength at 460 nm and excitation wavelength at 350 nm.

### Assessment of mitochondrial membrane potential (MMP) by flow cytometry and immunofluorescence

MMP assay kit (Beyotime, China) with JC-1 probe was used to measure MMP in PLC cells. Briefly, cells were seeded in six-well plates overnight and serum-starved for 24 h, then treated with various concentration of r*Cs*severin for 48 h. The cells were then washed with ice-cold PBS and incubated in a 5% CO_2_ humidified incubator at 37°C for 20 min after adding 1 ml of JC-1 working solution. The supernatant was then discarded and the cells were washed twice with JC-1 staining buffer. Next, 2 ml medium was added to each well and MMP was monitored using an inverted fluorescence microscope (Leica DMI4000B, Germany) and laser scanning confocal microscope (Zeiss LSM 710, Germany). The red JC-1 fluorescence was observed at 525 nm excitation (Ex)/590 nm emission (Em) and the green cytoplasmic JC-1 fluorescence was observed at 485 nm Ex/530 nm Em.

Quantitative changes of MMP at the early stage of cell apoptosis were measured by flow cytometry with JC-1 probe. After being incubated with 10, 20, 40 and 80 µg/ml of r*Cs*severin for 48 h, 1–5×10^5^ cells were harvested and resuspended with ice-cold PBS (1,500 rpm×5 min). Then, the cell suspensions were incubated with 0.5 ml JC-1 working solution in 0.5 ml DMEM for 20 min at 37°C. The staining solution was removed by centrifugation. The cells were washed with JC-1 (1×) washing buffer twice, then resuspended in 500 µl JC-1 (1×) staining buffer and detected by flow cytometer (Bechman Coulter Gallios, USA).

### Statistical analysis

All of the experiments were repeated at least three times. Experimental values were obtained from three independent experiments with a similar pattern and expressed as means ± standard deviation (SD). Statistical analyses were performed using SPSS software package 17.0. Data were analyzed by one-way analysis of variance (ANOVA) followed by least significant difference (LSD) for comparison between control and treatment groups. Significance was set at *p* value<0.05.

## Results

### Sequence analysis of *Cs*severin

The ORF of *Cs*severin contained 1077 base pairs (bp) encoding a protein of 358amino acids (predicted MW 40.88 kDa, pI 5.24). Blastx analysis showed that the deduced amino acid sequence was homologous to gelsolin of S*chistosoma mansoni, Schistosoma japonicum, Suberites domuncula, Echinococcus granulosus, Strongylocentrotus purpuratus* and *Hydra magnipapillata* with 54%, 65%, 50%, 65%, 48%, 47% identities respectively. The amino acid sequence had no N-terminal signal peptide or transmembrane domain. According to MotifScan and InterproScan prediction, there were three gelsolin domains (aa51–133, aa171–247, aa278–354) indicating that *Cs*severin might have similar role with gelsolin superfamily. Furthermore, we inferred that the location of putative actin binding surface of *Cs*severin was from 50 to 150 amino acids by Gene Ontology analysis (http://www.geneontology.org/). The nuclear magnetic resonance (NMR) derived structure of human (*Homo sapiens*) gelsolin (PRF: 225304) was used as the template to build a molecular model of *Cs*severin. The two proteins shared 36% identity among their gelsolin core domains and there was significant similarity between their tertiary structures (Figure S1).

### Phylogenetic relationships

*Cs*severin grouped very closely with *Schistosoma japonicum* (Figure S2), a parasite that increases the risk of HCC incident when associated with positive hepatitis B surface antigen [38]. The *Cs*severin was also closely relative to severin/gelsolin from *Echinococcus granulosus*, followed by *Dictyostelium discoideum*, but far from those of *H. sapiens* and *M. musculus*.

### Prokaryotic expression and purification of r*Cs*severin

The soluble r*Cs*severin was expressed with 6×His-tag in *E. coli* BL21 after induced by 1 mM IPTG at 37°C for 5 h. The purified recombinant protein showed a single band around 45 kDa (including His-tag sequence) in 12% SDS-PAGE, consistent with the predicted molecular mass (Figure S3, lane 7). The final protein concentration was 0.8 mg/L. The anti-r*Cs*severin serum was collected from immunized rat.

### Western blot analysis

**Figure 1:**
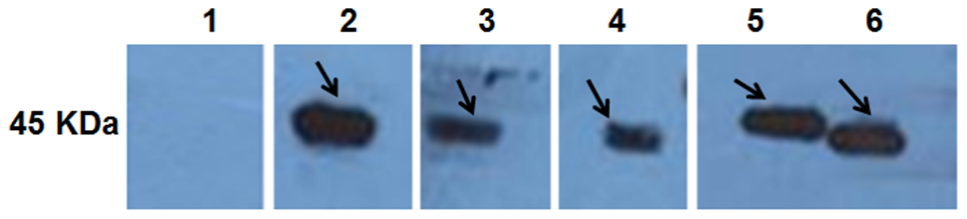
Western blotting of r*Cs*severin. r*Cs*severin reacted with sera from naive rats (lane 1), anti-His tag monoclonal antibody (lane 2), sera from *C. sinensis*-infected rats (lane 3), sera from rats immunized with the *Cs*ESP (lane 4), sera from rats immunized with r*Cs*severin (lane 5). *Cs*ESPs reacted with sera from rats immunized with r*Cs*severin (lane 6).

Purified r*Cs*severin could be recognized by rat anti-r*Cs*severin serum, anti-His tag monoclonal antibody, serum from *C. sinensis*-infected rat and serum from *Cs*ESPs-immunized rat at 45 kDa, while not blotted with serum from naïve rat. The *Cs*ESPs was probed by rat anti-r*Cs*severin serum at about 45 kDa. However, no band was detected by serum from naïve rat (Figure 1 lanes 1–6).

### Expression level of *Cs*severin at the stage of egg, metacercaria and adult worm of *C. sinensis*

**Figure 2:**
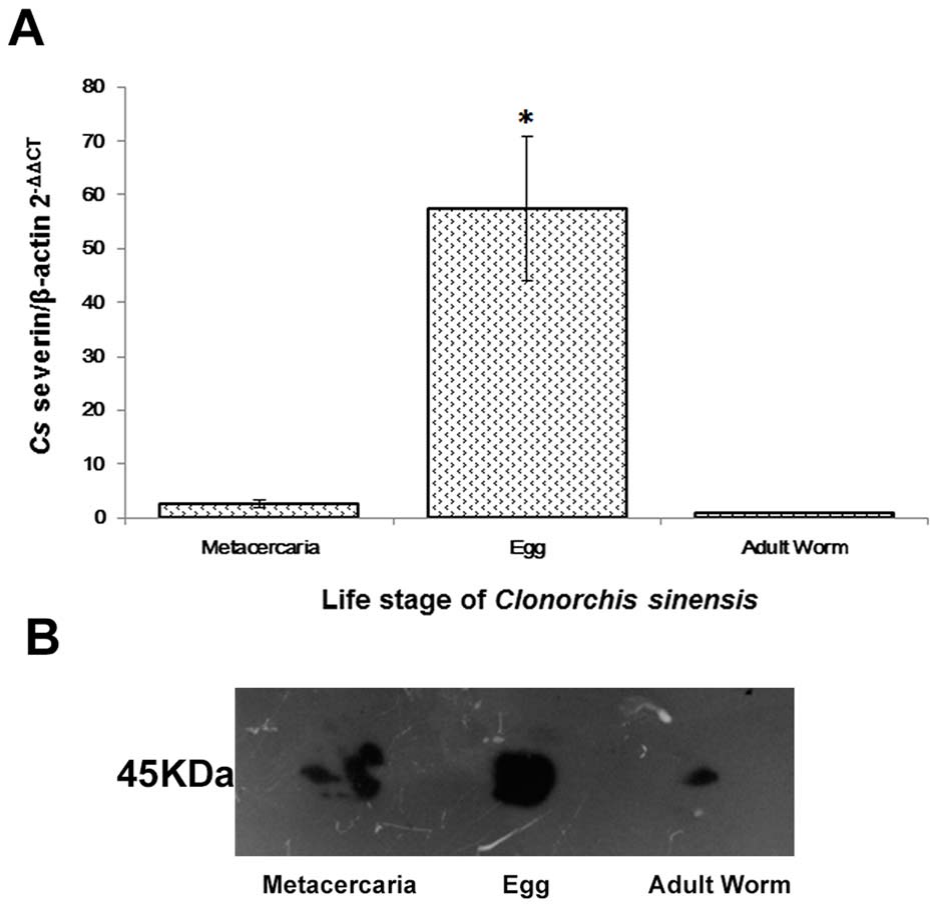
Gene/protein expression analysis of *Cs*severin at different life cycle stages of *C. sinensis*. (A) Quantitative real-time PCR analysis. The transcription levels of *Cs*severin at life stages of adult worm, metacercaria and egg were analyzed by means of the 2^−ΔΔCt^ ratio, with *Cs* β-actin serving as the internal standard. *: *p*<0.05, the transcription level of *Cs*severin in egg was statistically higher than that in adult worm and metacercaria. (B) Western blotting analysis. Thirty microgram of total proteins from each life cycle stage were subjected to SDS-PAGE and analyzed. Rat anti-r*Cs*severin serum was used as primary antibody at a dilution of 1∶100. The same dilution of pre-immune rat serum was used as a negative control, and no corresponding band was observed (not shown).

*Cs*severin were detected to express at life stage of metacercaria, egg and adult worm of *C. sinensis*, but at different levels. Statistically significant differences of transcripts were detected among metacercaria, egg and adult worm when normalized by β-actin. The transcription level of *Cs*severin in egg was about 60 times higher than that in adult worm (Figure 2A). The expression level of *Cs*severin was consistant with the transcriptional level. Egg has the highest expression level of *Cs*severin protein, followed by adult worm and metacercaria (Figure 2B).

### Immunolocalization of *Cs*severin in the adult worm and metacercaria of *C. sinensis*

**Figure 3:**
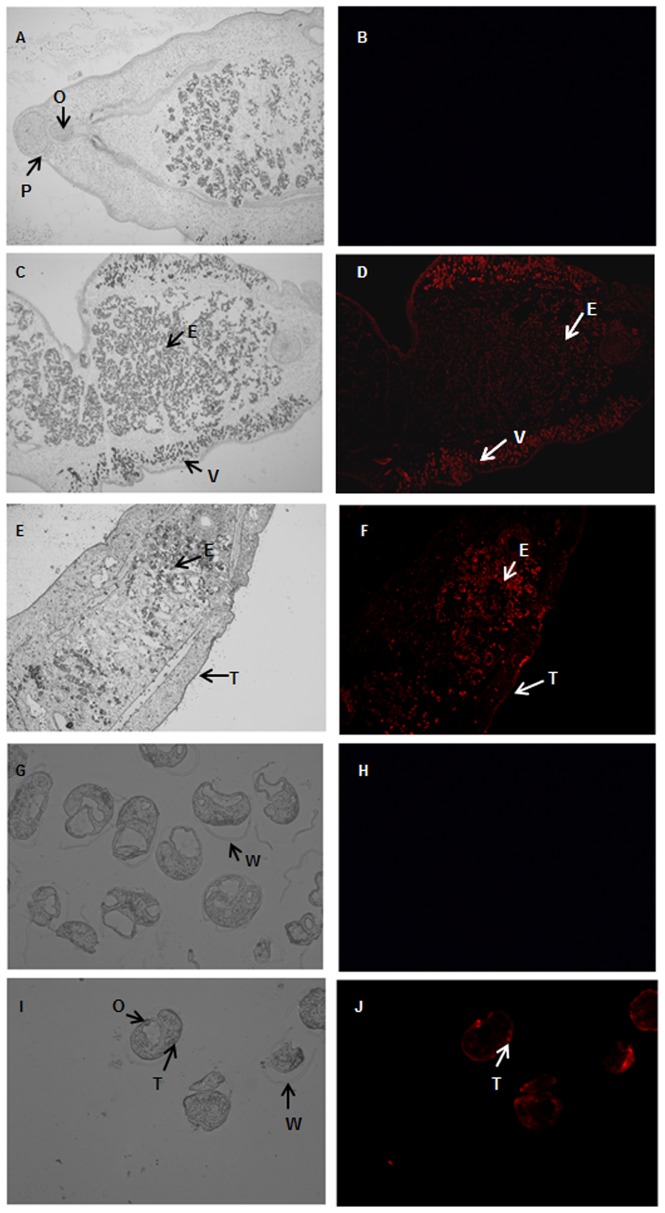
Immunolocalization of *Cs*severin in adult worm and metacercaria of *C. sinensis*. Rat anti-r*Cs*severin serum was used as primary antibody and red fluorescent Cy3-labeled goat anti-rat IgG as secondary antibody. Slides were observed under white light (pane A, C, E, G, I) or fluorescence microscope (pane B, D, F, H, J). No specific fluorescence was observed in pane B or H which was probed with serum from rat immunized with PBS as negative control. Intensive reddish-orange fluorescences were observed in vitellarium and intrauterine eggs of adult worm (pane D, F, ×50) and oral suck of metacercaria (pane J, ×200). Scattered fluorescences were detected in tegument of adult worm and metacercaria. V, vitellarium. O, oral sucker. T, tegument. E, eggs. W, cyst wall. P, pharynx.

The analysis of immunofluorescence localization by using rat anti-r*Cs*severin serum showed that in *C. sinensis* adult intensive fluorescences were observed in vitellarium while scattered fluorescences were detected in tegument. In metacercaria, specific fluorescences were only deposited in tegument. In addition, intensive fluorescences were presented in intrauterine eggs of adult worm (Figure 3D, F and J). By comparison, no specific fluorescence was detected either in adult worm or in metacercaria when treated with serum from naïve rat (Figure 3B, H).

### Analysis of circular dichroism (CD) spectrum

**Figure 4:**
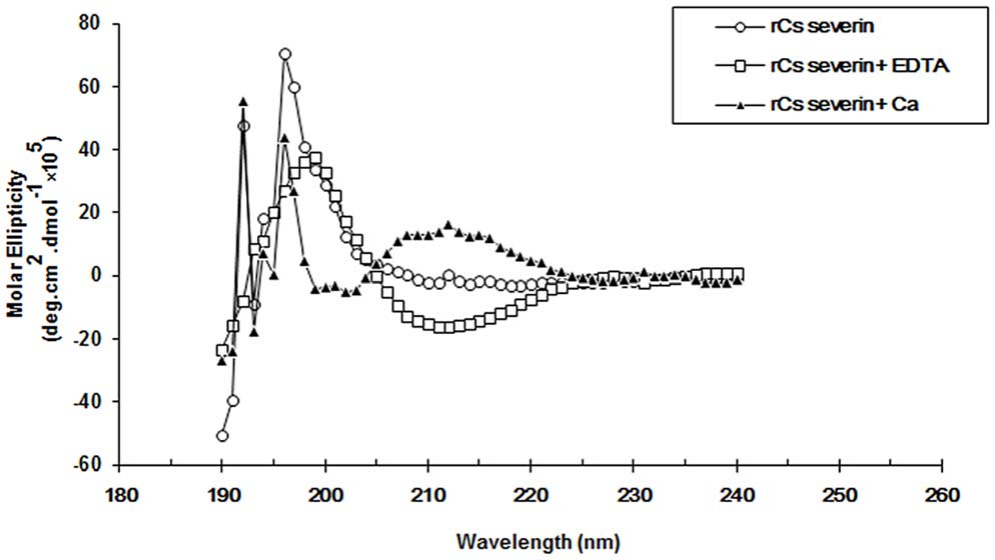
Far-UV CD spectra of r*Cs*severin in the absence and presence of Ca^2+^. Spectral changes of CD for PBS–r*Cs*severin solutions (1 µM in 10 mM) on the addition of the 1 µM calciumion (upper row) and 1 µM EDTA (lower row) conditions demonstrated r*Cs*severin could bind to Ca^2+^.

According to the profile of CD spectrum, the secondary structure of r*Cs*severin changed from the presence of Ca^2+^ shifted to the absence of Ca^2+^ (presence of EDTA) (Figure 4). With Ca^2+^, the secondary structure of r*Cs*severin contained 23.6% α-helix, 56.6% β-sheet, and 19.8% random loop. While with equivalent EDTA, it changed to 21.5% α-helix, 41.2% β-sheet, and 37.3% random loop. The conformation of the purified r*Cs*severin was between the two conditions with 24.6% α-helix, 49.9% β-sheet, 25.5% random loop. Ca^2+^-binding altered the conformation of EF-hand domain from α-helix to β-sheet. The purified r*Cs*severin partially combined Ca^2+^ during the processes of expression and purification. We showed that r*Cs*severin was easily to precipitate when calciumion was added into the solution, and can be resolved by adding EDTA.

### Interaction of r*Cs*severin with actin

**Figure 5:**
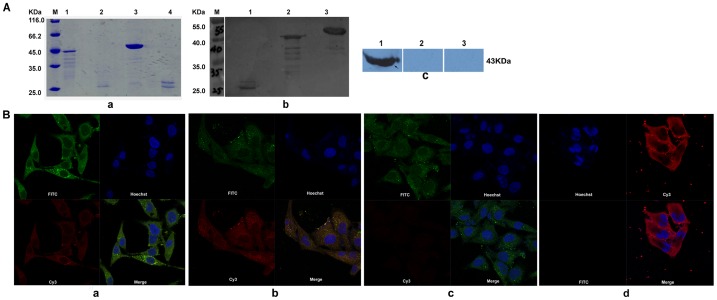
Interaction of r*Cs*severin with actin. (A) The bindings of r*Cs*severin to F-actin and its fragments were examined using gel overlay assay as described in materials and methods. In pane a, actin and its fragments were separated on 12.5% SDS-PAGE. Protein molecular weight markers (M), F-actin at 37°C for 1 h (lane 1), F-actin digested with 0.25% trypsin at 37°C for 1 h (lane 2), purified r*Cs*severin (lane 3), 0.25% trypsin at 37°C for 1 h (lane 4). In pane b, r*Cs*severin binding to F-actin and its fragments were examined using gel overlay assay. The membrane was incubated with r*Cs*severin then rat anti-r*Cs*severin serum. Protein molecular weight markers (M), F-actin fragments after digesting with 0.25% trypsin at 37°C for 1 h (lane 1), F-actin at 37°C for 1 h (lane 2), purified r*Cs*severin (lane 3). In pane c, the membrane was incubated with r*Cs*severin then rat anti-r*Cs*severin serum (lane 1), the membrane was incubated with BSA then rat anti-r*Cs*severin serum (lane 2), the membrane was incubated with r*Cs*severin then pre-immune rat serum (lane 3). (B) The bindings of r*Cs*severin to cytoskeletal actin filaments of PLC cells by immunocytochemistry. Pane a, cells were coated with r*Cs*severin before fixed with 4% paraformaldehyde and successively with rat anti-r*Cs*severin serum and mouse anti-F-actin monoclonal antibody, and then a mixture of FITC (green fluorescence) and Cy3 (red fluorescence)-labeled goat anti mouse/rat IgG. Pane b, cells were coated with r*Cs*severin after permeabilized with 0.3% Triton X-100 and successively with rat anti-r*Cs*severin serum and mouse anti-F-actin monoclonal antibody, and then a mixture of FITC (green fluorescence) and Cy3 (red fluorescence)-labeled goat anti mouse/rat IgG. Pane c, cells incubated with monoclonal anti-F-actin antibody without coated with r*Cs*severin as negative control. Pane d, cells were coated with r*Cs*severin before fixed with 4% paraformaldehyde, then incubated only with anti-r*Cs*severin serum after permeabilized with 0.3% Triton X-100 as another negative control. The images were taken under a LSM 710 Zeiss confocal microscope, with an oil immersion objetive (63×, 1.40 numerical aperture).

The binding of r*Cs*severin to F-actin and its fragments were examined using gel overlay assay as described above. After incubation with r*Cs*severin, F-actin and its fragments were blotted by anti-r*Cs*severin serum (Figure 5A, pane b, lane 1–2 and pane c, lane 1). While incubation with BSA or without r*Cs*severin (Figure 5A, pane b, lane 2–3), F-actin couldn't be probed by anti-r*Cs*severin serum. Whether PLC cells were incubated with r*Cs*severin before or after fixation and permeabilization, both the green fluorescence (FITC–conjugated affinipure goat anti-mouse IgG reacted with anti-F-actin monoclonal antibody) and the red fluorescence (Cy3–conjugated affinipure goat anti-rat IgG reacted with anti-r*Cs*severin serum) were observed. The locations of green fluorescence were mostly coincident with those of the red fluorescence (Figure 5B, pane a and b). There was no red fluorescence or green fluorescence in negative control group (Figure 5B, pane c and d). Thus, we suspected that r*Cs*severin might enter into PLC cells and bind to actin.

### Apoptosis assay

**Figure 6:**
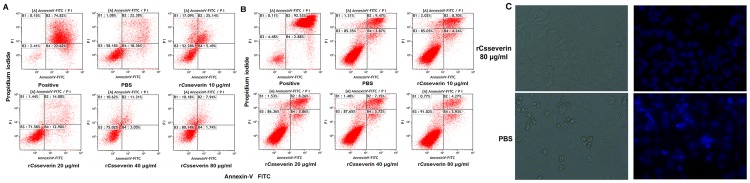
Apoptosis inhibition of PLC cells with r*Cs*severin in a concentration-dependent manner. (A) Flow cytometry analysis of PLC cells treated with PBS, apoptosis inducer (positive control) and 10, 20, 40, and 80 µg/ml r*Cs*severin for 48 h after induction of spontaneous apoptosis. Representative dot plots of cell apoptosis were showed after AnnexinV/PI dual staining. Apoptotic rate was represented as a percentage of total cell populations. The proportion of dead cells (Annexin V−/PI+), live cells (Annexin V−/PI−), early apoptotic cells (Annexin V+/PI−) and late apoptotic/necrotic cells (Annexin V+/PI+) was respectively measured for comparison. (B) Flow cytometry analysis of L-02 cells with the same treatment. (C) Morphologic changes in apoptotic PLC cells. Following treatment with PBS (negative control) or 80 µg/ml r*Cs*severin for 48 h, apoptotic nuclei were condensed and brightly stained with Hoechst 33258 then nuclear morphology was photographed and visualized with a Leica DMI4000B (Magnification×400). Each experiment was performed in triplicate.

To identify the effect of r*Cs*severin on PLC cells, we tested the total percentage of Annexin V+/PI− and Annexin V+/PI+ cells by flow cytometry. As shown in Figure 6A, incubation of PLC cells with different dosages of r*Cs*severin (10, 20, 40, and 80 µg/ml) for 48 h after induced spontaneous apoptosis by serum-starved for 24 h decreased the percentage of Annexin V+/PI− and Annexin V+/PI+ cells in a dose-dependent manner (30.63, 26.98, 14.36, and 9.68%, respectively), as compared to the PBS-treated controls, which showed 40.74% Annexin V+/PI− and Annexin V+/PI+ cells. The results showed that r*Cs*severin exhibited potent anti-apoptosis activity on PLC cells in concentration-dependent manner. We also tested the effect of r*Cs*severin on human normal hepatocyte L-02 cells. No significant decrease of Annexin V+/PI− and Annexin V+/PI+ cells was observed (Figure 6B).

We also compared the morphology of PLC cells in the presence of 80 µg/ml r*Cs*severin to that of PBS-treated cells under the inverted phase-contrast microscopy. Hoechst staining of PBS-treated cells after induced spontaneous apoptosis by serum-starved for 24 h revealed marked morphological changes, such as cell shrinkage, vesicular degeneration, threadlike morphology, nuclear condensation, and nuclear fragmentation, which are typical features of apoptotic cell death. While morphological changes of the PLC cells in presence of 80 µg/ml r*Cs*severin after treatment with serum-starved for 24 h were not significant (Figure 6C).

### Recovery of the mitochondrial membrane potential (MMP) in r*Cs*severin treated PLC cells

**Figure 7:**
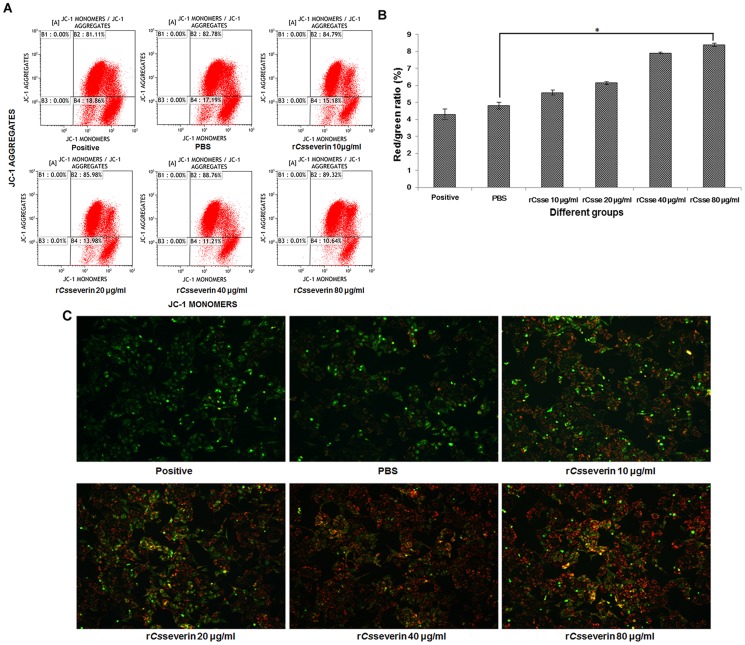
Effect of r*Cs*severin on mitochondrial membrane potential in PLC cells. (A) Following treatment with PBS (negative control) or 80 µg/ml r*Cs*severin for 48 h, representative dot plot showed the changed MMP by flow cytometry after the labeling of fluorescent probe with JC-1. Reduction of mitochondrial membrane potential was demonstrated by the change in JC-1-derived fluorescence from red (JC-1 aggregates, representing high potential) to green (JC-1 monomer, representing low potential). (B) The quantitative MMP from each group was marked by the intensity ratio of red fluorescence over green fluorescence by flow cytometry. The data are expressed as mean ± SD from three independent experiments. * means *p*<0.05, there is statistic difference (ANOVA/Dunnett's T3 test) between 80 µg/ml r*Cs*severin group and PBS group (negative control). (C) Typical fluorescence photomicrograph of *in situ* JC-1 staining output by laser scan confocal microscopy (Magnification×100).

To further investigate the molecule events triggered by r*Cs*severin inhibition, we measured MMP in the PLC cells by using flow cytometry and JC-1 staining *in situ*. The decline of MMP is considered as a symbolic event of early cellular apoptosis. Changes in MMP can be assessed by monitoring JC-1, which accumulates in mitochondria forming red fluorescent aggregates at high membrane potential and exits mainly in cytosol forming a green fluorescent monomer, presenting a collapse of the membrane [39]. In our study, r*Cs*severin-treated cells showed reduction of green fluorescence and production of an obvious red fluorescence. The treatment of r*Cs*severin recovered the MMP in a concentration-dependent manner (Figure 7, A and B), as indicated by an increase of red (JC-1 aggregates)/green (JC-1 monomers) ratio. At 48 h, the percentage of 80 µg/ml r*Cs*severin and PBS treated PLC cells which emitted green fluorescence was 15.42 and 9.63%, respectively, indicating the recovery of mitochondrial membrane depolarization. The PLC cells that treated with apoptosis introducers exhibited mitochondrial green fluorescence with little red fluorescence, suggesting the cells in depolarization state. The red fluorescence in PLC cells increased, as monitored by *in situ* JC-1 staining, after the treatment of 10, 20, 40, 80 µg/ml r*Cs*severin as compared with the PBS group (Figure 7C).

## Discussion

In the present study, we identified that *Cs*severin, which expressed at life stage of egg, metacercaria and adult worm was a component of *Cs*ESPs. We also demonstrated its ability of binding with calciumion and actin filaments. Furthermore, co-incubation of PLC cells with r*Cs*severin *in vitro* led to an inhibition of apoptosis induced by serum-starved for 24 h, by using morphological analysis of PLC, detection of the apoptosis-associated change of mitochondrial membrane potential as well as Annexin V/PI apoptosis assay. We inferred that r*Cs*severin may play an intracellular protective role via preventing apoptotic mitochondrial changes (the loss of mitochondrial membrane potential), just like endogenous human gelsolin did [40].

Gelsolin family is found in a diverse range of organisms including bacteria, invertebrates, plants, primates, rodents and vertebrates. The superfamily in mammals consists of seven different proteins: gelsolin, adseverin, villin, capG, advillin, supervillin and flightless I. All of them contain three or six homologous repeats of a domain named gelsolin-like (G) domain [41]. Bioinformatics analysis showed that *Cs*severin comprised three gelsolin homology domains, calciumion and actin binding motifs. The amino acid sequence of *Cs*severin shared 36% identity with that of human gelsolin, but there was significant similarity between their tertiary structures. Our phylogenetic analysis suggested that a majority of gelsolin proteins do not form clades based on taxonomic groupings but rather group according to protein functions. The individual gelsolin domains from human gelsolin form distinct clades with homologues from other species, supporting the notion that these proteins have evolved to perform distinct functions in different organisms.

Increased Ca^2+^ influx through voltage-dependent Ca^2+^ channels is the major determinant of cell injury following excitotoxicity [42], [43]. The activity of these channels is modulated by dynamic changes in the actin cytoskeleton [44], [45], which may occur, in part, through the actions of gelsolin [46]. We obtained soluble and stable r*Cs*severin. CD measurements actually showed that r*Cs*severin could bind to calciumion. It has been documented that gelsolin family is of actin-regulatory function [47]. Cytoskeletal actin filaments are dynamic structures that form membranous networks interacting with cell surface receptors and intracellular effectors [48], [49]. Gel overlay and immunocytochemistry assay indicated the binding activity of r*Cs*severin.

Gelsolin expression in certain tumors correlates with poor prognosis and therapy-resistance. *In vitro*, human gelsolin has anti-apoptotic and pro-migratory functions and is critical for invasion of some types of tumor cells [50], [51], [52], [53]. We found that gelsolin was highly expressed at tumor borders infiltrating into adjacent liver tissues [54]. In Jurkat lymphoblastoid T-cell line, gelsolin has been shown to inhibit apoptosis, and the overexpression of gelsolin inhibits the loss of mitochondrial membrane potential and cytochrome c release from mitochondria [55]. Additionally, in several models of neuronal cell death, endogenous gelsolin has been demonstrated that has an anti-apoptotic property which correlates to its dynamic actions on the cytoskeleton mediated by inhibition of mitochondrial permeability transition [56].

Here we also showed that r*Cs*severin could cause obvious apoptotic inhibition in the human HCC cell line. Flow cytometry was used to evaluate r*Cs*severin-inhibited apoptosis after dual staining of cells with AnnexinV and PI. Due to that Annexin V binding is based on the transposition of phosphatidyl serine from the inner to the outer face of the cell membrane during the early stages of apoptosis [57]. This method has been widely used to discriminate between normal cells (AnnexinV−/PI−), early apoptotic cells AnnexinV+/PI−), late apoptotic cells (AnnexinV+/PI+), and necrotic cells (AnnexinV−/PI+). Compared with PBS-treated group (negative control), there were less typical apoptotic changes in r*Cs*severin-treated PLC cells after induced spontaneous apoptosis by serum-starved for 24 h in morphology analysis. We also measured the changes in mitochondrial membrane potential (MMP) using a JC-1 probe that gives a red fluorescence when MMP is high and green fluorescence when MMP is low that occurs in early apoptosis cells. We found that interact directly with r*Cs*severin led to the recovery of mitochondrial membrane potential in PLC cells.

Moreover, r*Cs*severin could be probed by sera from rat infected with *C. sinensis* besides anti-*Cs*ESPs serum that confirmed *Cs*severin was a molecular of *Cs*ESPs. Although it is still unclear about the mechanism of uptake or internalization of *Cs*ESPs by host cells, internalized *Cs*ESPs could play roles in the interaction between the host and parasite. These data demonstrated that *Cs*severin, as an anti-apoptotic molecule to carcinoma cell, might be a pathogenic factor in *Cs*ESPs, contributing to the development of a pro-tumorigenic environment that was conductive to HCC.

Tissue-specific distribution of *Cs*severin in muscular locations such as teguments of adult worm and metacercaria, as well as its actin binding activity, we inferred that *Cs*severin might involve in regulating the contraction of smooth muscle and movement of worm body [58], [59], [60]. What was more, relative high transcript/protein level of *Cs*severin at egg stage was consist with its intensive immunolocalization in the intrauterine eggs of adult worm. As a food-borne parasite, *C. Sinensis* adult lives in the bile ducts of the host and the worm releases a mass of eggs and ESPs, so that *Cs*severin exists in parasitism circumstance sustainedly and takes a part in the interaction between the host and parasite.

Overall, we presented the molecular characteristics of *Cs*severin, a molecule of *Cs*ESPs. Recombinant *Cs*severin (r*Cs*severin) could bind to Ca^2+^ and cytoskeletal actin filaments and cause obvious apoptotic inhibition in human HCC cell line. By promoting apoptosis inhibition, *Cs*severin might exacerbate the process of HCC patients combined with *C. sinensis* infection. More experiments should be further conducted. The current study may provide a novel insight in understanding the pathogenesis of carcinoma associated with the infection of *C. sinensis*, which was an inducing factor that cannot be ignored in the process of the development of primary hepatic carcinoma. Since gelsolin has actin-regulatory functions, modulation of the actin network might be responsible for the inhibition of apoptosis, the actin cytoskeleton may be a target to protect from apoptosis [61]. The anti-apoptotic mechanism of *Cs*severin are worthy of studying in the future.

## Supporting Information

Figure S1 **Sequence analysis of severin of** ***Clonorchis sinensis*** **(** ***Cs*** **severin).**
(DOC)

Figure S2 **Neighbor joining phylogenetic tree for the gelsolin core domains from a range of phyla.**
(DOC)

Figure S3 **Prokaryotic expression and purification of r** ***Cs*** **severin by 12% SDS-PAGE.**
(DOC)
