# Expression of Concern: Molecular Characterization of Severin from *Clonorchis sinensis* Excretory/Secretory Products and Its Potential Anti-apoptotic Role in Hepatocarcinoma PLC Cells

**DOI:** 10.1371/journal.pntd.0012043

**Published:** 2024-03-19

**Authors:** The PLOS Neglected Tropical Diseases Editors

The following concerns were raised about Figs 3 and 5 of this article [1]:

- In Fig 5Ac, lanes 2 and 3 appear similar.
- When levels are adjusted to visualize background, there is no visible evidence that Figs 3B, 3H and 5Bd FITC panels show the same fields of view as the corresponding white light microscopy (Fig 3) and Hoechst/Cy3/Merge (Fig 5Bd) panels.

Corresponding author YH stated that the original data underlying the results reported in this article are no longer available, and so the above issues cannot be resolved.

In addition, the study reports the use of “human normal hepatocyte L-02” cells in flow cytometry experiments shown in Fig 6B. Following the publication of this article [1], the L-02 cell line was identified as contaminated and shown to be a HeLa derivative instead [2]. Therefore, the results statements in [1] about effects of r*Cs*severin on human normal hepatocyte cells are not supported, although this may not critically impact the PLC cell results reported in Fig 6A.

The *PLOS Neglected Tropical Diseases* Editors issue this Expression of Concern to inform readers of these unresolved issues. Readers should interpret the results of the affected figures with caution.

## References

[1] Chen X, Li S, He L, Wang X, Liang P, Chen W, et al. (2013) Molecular Characterization of Severin from *Clonorchis sinensis* Excretory/Secretory Products and Its Potential Anti-apoptotic Role in Hepatocarcinoma PLC Cells. PLoS Negl Trop Dis 7(12): e2606. 10.1371/journal.pntd.0002606 24367717 PMC3868641

[2] Ye F, Chen C, Qin J, Liu J, Zheng C (2015) Genetic profiling reveals an alarming rate of cross-contamination among human cell lines used in China. FASEB J 29(10):4269–72. doi: 10.1096/fj.14-266718 26116706

