# Supplementary material for: Molecular Characterization of Severin from Clonorchis sinensis Excretory/Secretory Products and Its Potential Anti-apoptotic Role in Hepatocarcinoma PLC Cells
Source: PLoS Negl Trop Dis. 2013 Dec 19;7(12):e2606. doi: 10.1371/journal.pntd.0002606 (PMC3868641; doi:10.1371/journal.pntd.0002606)
Supplement: Figure S1 — Sequence analysis of severin of Clonorchis sinensis ( Cs severin). (DOC) [file pntd.0002606.s001.doc]

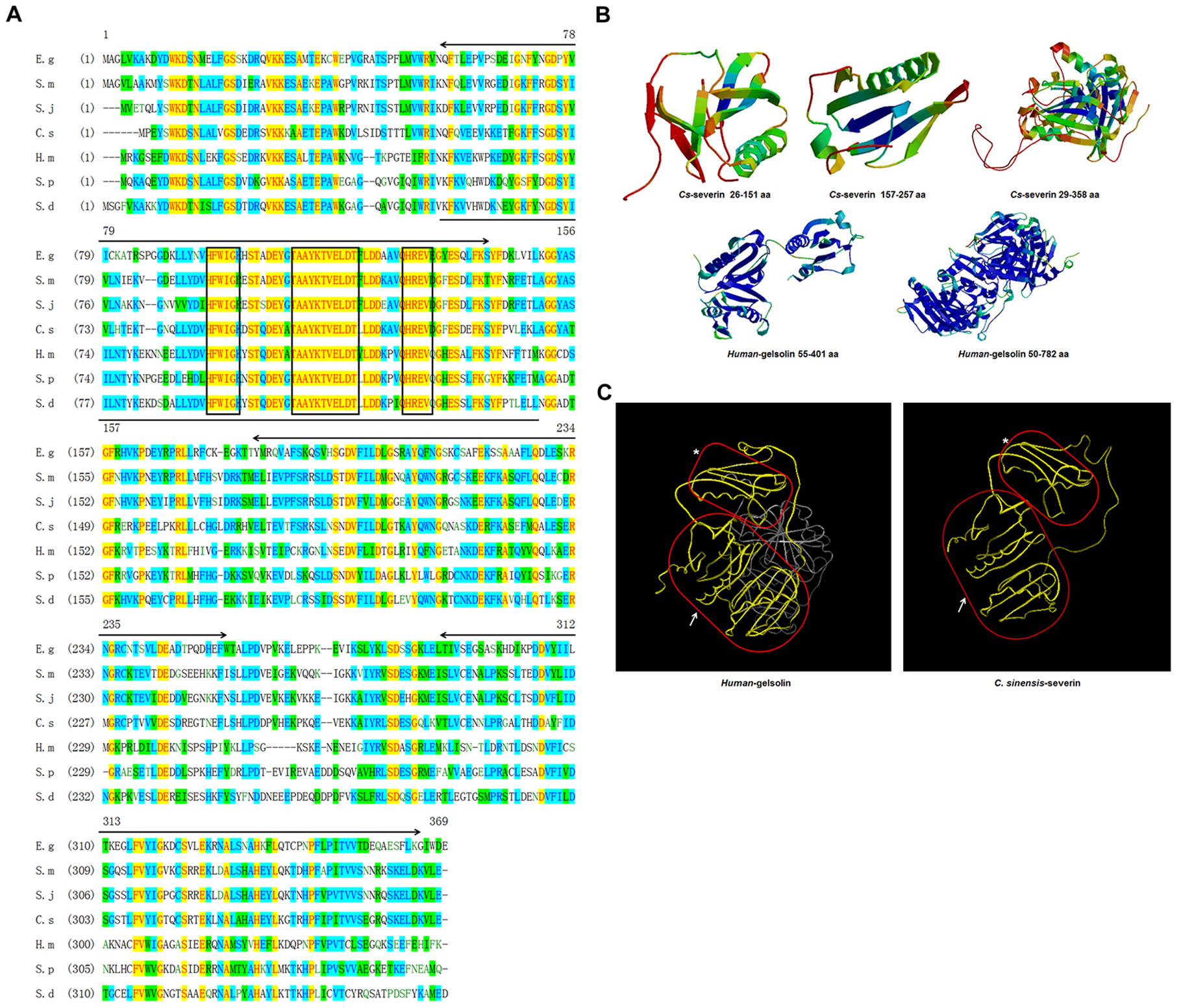


**Figure S1. Sequence analysis of severin of *Clonorchis sinensis* (*Cs*severin)**. (A). Multiple sequence alignments of *Cs*severin and gelsolin domains from other organisms. The gelsolin domains were illustrated in double arrow. Conserved motifs were illustrated in boxes and the locations of putative actin binding surfaces were shown in overline. Amino acid sequence of *Cs*severin shares 54, 65, 50, 65, 48, 47% identities with that of *Schistosoma mansoni* (XP_002572342.1), *Schistosoma japonicum* (CAX82644.1), *Echinococcus granulosus* (AAK15753.1), *Strongylocentrotus purpuratus* (XP_792912.2), *Suberites domuncula* (CAC87029.1), *Hydra magnipapillata* (XP_002155137.2). (B). Molecular model of the gelsolin core domain of *Cs*severin compared with the nuclear magnetic resonance structure of human gelsolin (PRF: 225304). (C) Comparision of putative spatial structure of *Cs*severin with that of human gelsolin (PRF: 225304). The aligned domains were marked in yellow color and Unmatched domain in C-terminus of human gelsolin was presented in gray. The similar structures were illustrated in red boxes respectively signed with arrows and asterisk.
