# Supplementary material for: Molecular Characterization of Severin from Clonorchis sinensis Excretory/Secretory Products and Its Potential Anti-apoptotic Role in Hepatocarcinoma PLC Cells
Source: PLoS Negl Trop Dis. 2013 Dec 19;7(12):e2606. doi: 10.1371/journal.pntd.0002606 (PMC3868641; doi:10.1371/journal.pntd.0002606)
Supplement: Figure S2 — Neighbor joining phylogenetic tree for the gelsolin core domains from a range of phyla. (DOC) [file pntd.0002606.s002.doc]

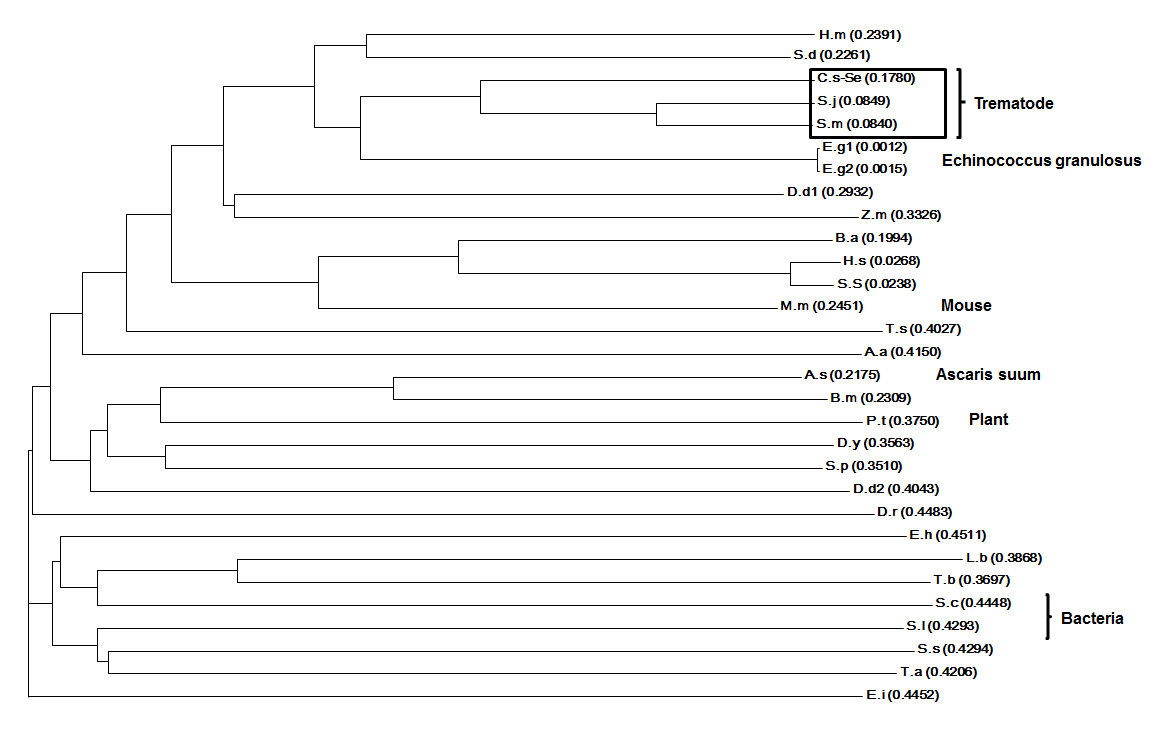


**Figure S2. Neighbor joining phylogenetic tree for the gelsolin core domains from a range of phyla.** Amino acid sequences of serverin/gelsolin from other species were included. The sequences were as follows: *Streptomyces lavendulae* (ABI22147.1), *Streptomyces sp. C* (EFL19489.1), *Leishmania braziliensis* (XP_001564461.1), *Hydra Magnipapillata* (XP_002155137.2), *Dictyostelium discoideum*1 (AAA33250.1), *Dictyostelium discoideum*2 (AAB36957.1), *Drosophila yakuba* (AAL31725.1), *Homo sapiens* (1211330A), *Danio rerio* (NP_001030338.2), *Mus musculus* (NP_033535.2), *Gallus gallus* (NP_001004406.1), *Theileria annulata* (XP_953849.1), *Strongylocentrotus purpuratus* (ABE27960.1), *Brugia malayi* (XP_001898075.1), *Aedes aegypti* (XP_001655525.1), *Trypanosoma brucei* (AAX79053.1), *Schistosoma japonicm* (CAX82644.1), *Schistosoma mansoni* (XP_002572342.1), *Caenorhabditis brenneri* (ACD86923.1), *Ascaris suum (*ADY47192.1), Echinococcus granulosus (AAK15753.1), Bos Taurus (BAA05548.1), *Sus scrofa* (CAA32077.1), *Suberites domuncula* (CAC87029.1), *Populus trichocarp* (ABK95801.1), *Zea mays* (ACL53623.1).
