# Supplementary material for: Molecular Characterization of Severin from Clonorchis sinensis Excretory/Secretory Products and Its Potential Anti-apoptotic Role in Hepatocarcinoma PLC Cells
Source: PLoS Negl Trop Dis. 2013 Dec 19;7(12):e2606. doi: 10.1371/journal.pntd.0002606 (PMC3868641; doi:10.1371/journal.pntd.0002606)
Supplement: Figure S3 — Prokaryotic expression and purification of r Cs severin by 12% SDS-PAGE. (DOC) [file pntd.0002606.s003.doc]

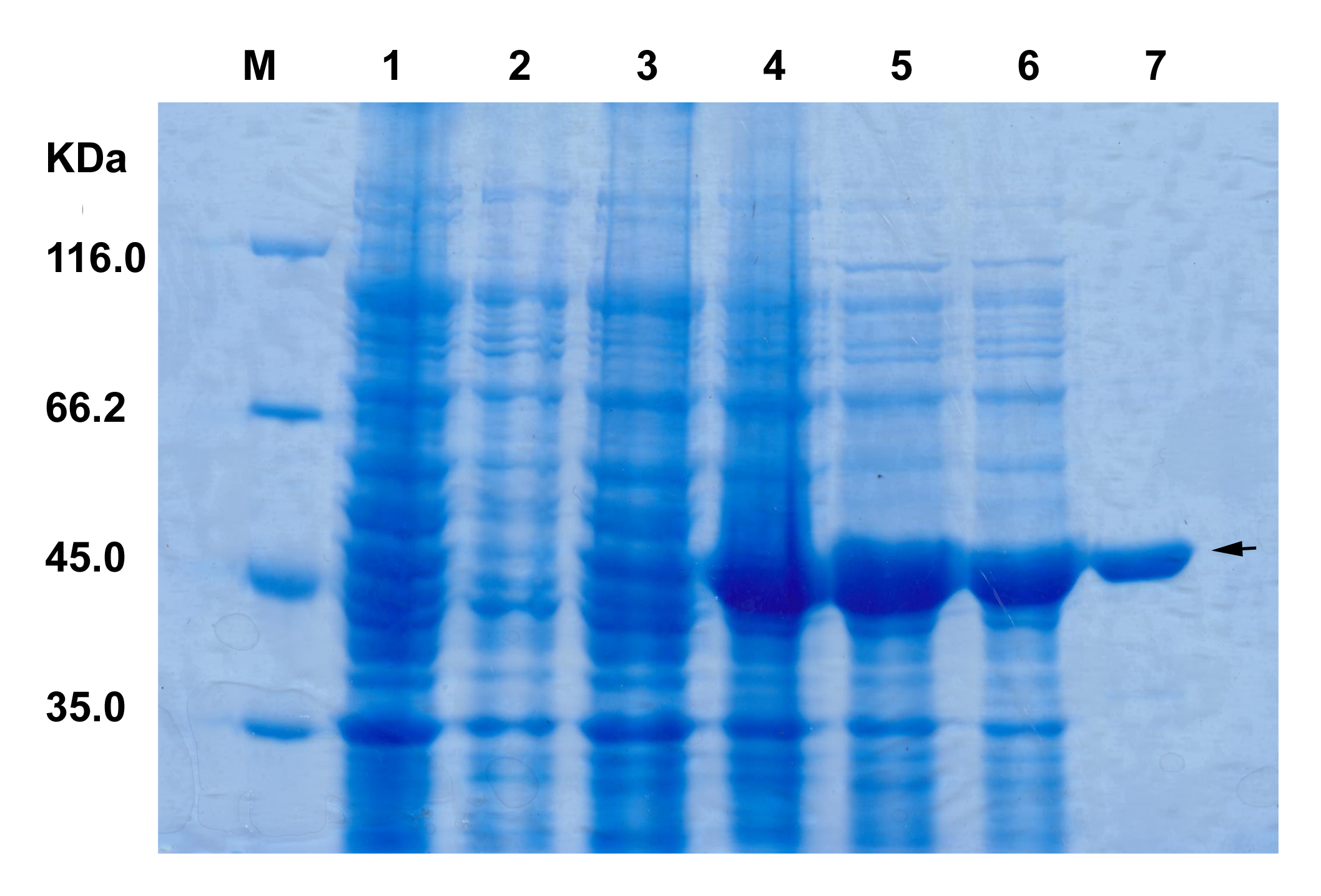


**Figure S3.** **Prokaryotic expression and purification of r*Cs*severin by 12 % SDS-PAGE.** r*Cs*severin expressed in *E. coli* with IPTG induction was purified by His-band resin chromatography. Protein molecular weight markers (M), lysate of *E. coli* with pET-28a(+) without IPTG induction (lane 1) and with IPTG induction (lane 2), lysate of *E. coli* with pET28a(+)-r*Cs*severin before induction (lane 3) and after induction (lane 4), supernatant (lane 5), and sediment (lane 6) of induced pET28a(+)-r*Cs*severin, purified r*Cs*severin (lane 7).
